# Daily 10 mg rivaroxaban as a therapy for ventricular thrombus related to left ventricular non-compaction cardiomyopathy: A case report

**DOI:** 10.1097/MD.0000000000009670

**Published:** 2018-01-26

**Authors:** Huan Sun, Qini Zhao, Yanjing Wang, Robert Lakin, Haiyan Feng, Xingyu Fan, Huiling Luo, Dongmei Gao, Lin Liu, Yuquan He, Ping Yang

**Affiliations:** a Cardiology Department; b Radiology Department, China-Japan Union Hospital of Jilin University, Changchun, China; c Department of Exercise Sciences, University of Toronto, Toronto, Ontario, Canada; d Ultrasound Department, China-Japan Union Hospital of Jilin University, Changchun, China.

**Keywords:** anticoagulation, LVNC, NOACs

## Abstract

**Rationale:**

Left ventricular non-compaction cardiomyopathy (LVNC) is a rare heart disorder related to thrombosis. Anticoagulant therapy is suggested for the treatment of this disease. The success of the novel oral anticoagulant rivaroxaban as a treatment option for this disorder is unclear.

**Patient concerns:**

A 43-year-old man who felt dizzy at rest was found to have an intraventricular thrombus.

**Diagnoses:**

The thrombus was confirmed by echocardiography. And LVNC was diagnosed by cardiac magnetic resonance (CMR) and echocardiography.

**Interventions:**

He was prescribed a low dose (10 mg daily) of rivaroxaban as treatment.

**Outcomes:**

After 3 months, the thrombus diminished, and the manifestation disappeared.

**Lessons:**

Low dose of rivaroxaban may serve as a viable option for anticoagulation therapy in LVNC patients, with large clinical trials needed to determine the best course of treatment.

## Abstract 2

Supplemental Digital Content is available in the text

## 1 Introduction

Left ventricle non-compaction cardiomyopathy (LVNC) is characterized by “spongy” LV myocardium, abnormal trabeculation, and deep intratrabecular recesses resembling the endocardial surfaces of an early embryonic heart.^[1,2]^ Though the incidence and prevalence of LVNC is unclear^[3]^ and LVNC has been considered a relative rare disorder, it has been reported as the third commonly diagnosed cardiomyopathy.^[3,4]^ Moreover, the clinical presentation of LVNC is highly variable, manifesting as end-stage heart failure or associated with arrhythmogenesis or thromboembolic events.^[5]^ The clinical diagnosis is based mainly on cardiac imaging including echocardiography, cardiac computed tomography (CT), as well as cardiac magnetic resonance (CMR), which are useful to identify and characterize the hyper-trabeculation of the heart.^[6,7]^ Lacking a clear etiology, the therapy typically consists of a combination of heart failure, anti-arrhythmia, and anti-thrombosis treatment.^[3]^

The thromboembolic risks associated with LVNC are well-established,^[8,9]^ which necessitates the need for antiplatelet and/or anticoagulation therapy.^[10]^ However, the low prevalence of LVNC renders the efficacy of anti-thrombolytic therapy in the context of LVNC unclear. The majority of case reports have adopted a warfarin anticoagulant treatment strategy in LVNC.^[11]^ As the era of novel oral anticoagulations (NOACs) has arrived, NOACs may offer a safer and more convenient alternative therapeutic approach in LVNC patients. Specifically, rivaroxaban have been shown to provide effective thromboprophylaxis and a reduced risk of recurrent thromboembolism, without a significant increase in bleeding rates.^[12]^ Moreover, the benefits of rivaroxaban have been shown at low doses (10 mg) that are typically associated with prophylaxis.^[13]^ While rivaroxaban has proven effective in thrombus treatment postinfarction^[14]^ at doses >15 or 20 mg daily, there are no reports or clinical trials studying whether low-dose rivaroxaban may be effective at managing the thromboembolic risks associated with LVNC.

## 2 Case description

A 43-year-old Asian male, with a 71-kg bodyweight and normal renal function, presented in the Cardiology Department at the China-Japan Union Hospital of Jilin University. He was found to have a mass in the left ventricle in May 2017 during his examination. The patient felt dizzy at rest without falling, spinning, or tinnitus. Dizziness persisted for 1 day and a routine echocardiographic examination discovered “suspected cardiac masses” in left ventricle, which led admission to our hospital. The patient suffered a brain injury 20 years prior, which caused an old trauma in a brain MRI examination without a novel ischemic lesion. The physical examination showed no abnormal signs on the heart.

**Figure 1:**
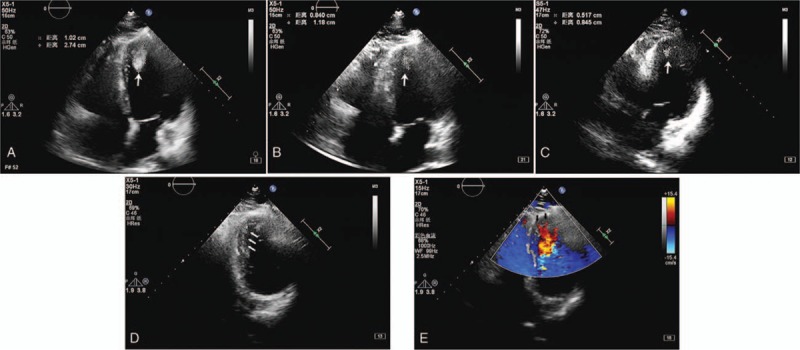
Serial echocardiographic images in the identification of a left ventricular thrombosis-like mass. (A) Identification of a thrombus at the left ventricular apex during the initial echo examination, leading to the initialization of rivaroxaban (10 mg daily) treatment. Crossed lines surrounded the thrombus-like mass, which is also indicated by an arrow. (B) After 15 days of treatment, the size of the thrombus was reduced. (C) At 45 days of anticoagulation therapy, the size of the thrombus was further reduced. (D) The thrombus had diminished after 3 months, revealing suspected increased intratrabacular recesses of the heart (blue arrows). (E) Doppler imaging revealed a “flushed” pattern of blood flow into the myocardium.

In our center, the echocardiography found a thrombosis-like mass in left ventricle, which was confirmed by cardiac acoustic contrast, and the patient presented with diminished systolic function characterized by an ejection fraction (EF) as low as 43%. The size of the mass was measured as 1.10 cm × 2.43 cm (Fig. 1A). Moreover, coronary arteriography showed no significant stenosis. As the patient was reluctant to undergo the repeated blood tests for warfarin treatment and bleeding risk, he was prescribed rivaroxaban 10 mg orally once daily.

**Figure 2:**
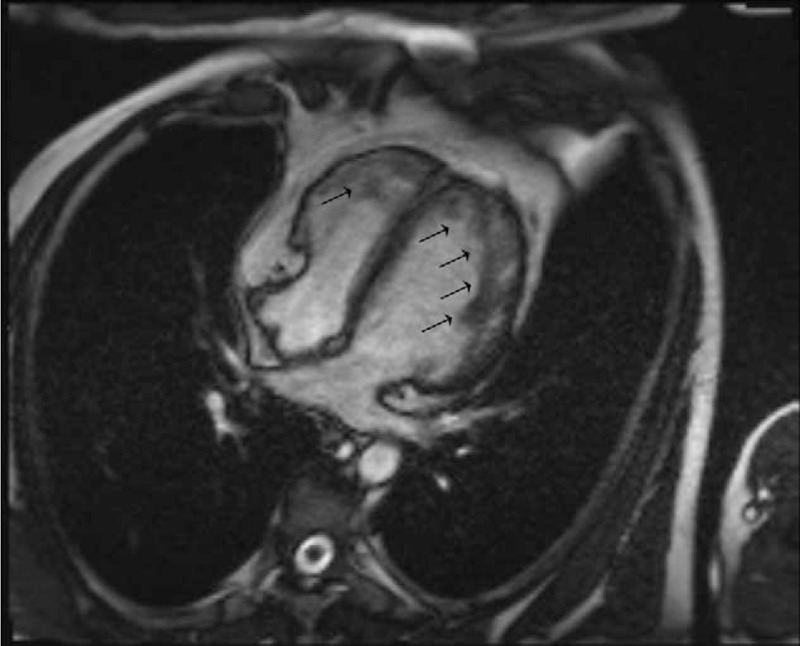
Cardiac magnetic resonance image showing evidence of ventricular non-compaction. Arrows indicate the non-compacted ventricle. The ratio between non-compaction to compaction of the ventricular myocardium is >2.3 at the end of diastole, which is one of the diagnostic criteria for LVNC.^[15]^

During the follow-up after 15 days, 45 days and 3 months, the size of the thrombus got decreased and vanished at the 3-month follow-up (Fig. 1B–D). As the thrombus was gone, echocardiographic assessment revealed a suspected left ventricular non-compaction cardiomyopathy (Fig. 1D and E). A follow-up cardiac magnetic resonance exam found hyper-trabeculation of the myocardium at apex and free wall (Fig. 2 and Online appendix A and B), which confirmed the diagnosis of LVNC. Twenty four hours Holter monitoring suggested evidence of a non-sustained ventricular tachycardia. Hence, the patient was given 10 mg oral rivaroxaban once daily to prevent thrombosis as well as beta blocker to improve cardiac function and prevent heart attack.

## 3 Discussion

LVNC is a rare heart disease. Although the LVNC is reported as the third commonly diagnosed cardiomyopathy, the prevalence is only about 0.05% in adults.^[3]^ The diagnosis of LVNC is based on imaging and clinic features. The manifestations of LVNC include heart failure, arrhythmia, and thrombosis. Though we observed a decreased in systolic function, our patient had no significant symptoms of heart failure, such as shortness of breath or edema, which made the diagnosis of LVNC preferable to non-compaction secondary to dilated cardiomyopathy. Moreover, while we discovered a ventricular arrhythmia by Holter monitoring, the arrhythmia was not sustained and caused no independent symptoms. Hence, beta-blocker therapy was initiated to treat both the arrhythmia and heart failure. The symptoms in our patient were most likely caused by thrombosis and transient ischemia, with evidence of recovery observed after anti-thrombosis therapy.

As the thromboembolic risk in LVNC is well known, the choice of anti-thrombosis therapy in these patients is clear.^[10]^ However, limited evidence exists to determine the correct course of anti-thrombotic medication options partly due to the low prevalence LVNC. Aspirin is one documented anti-platelet medication option in LVNC patients.^[16]^ In our patient, the finding of thrombosis in left ventricle made systemic anticoagulation therapy a preferable choice.^[17]^ To the best of our knowledge, this is the first report on using rivaroxaban in LVNC management. Novel oral anticoagulants (NOACs) such as rivaroxaban have shown therapeutic advantages to the commonly administered warfarin during systemic anticoagulation, including improved safety, reduced risk of bleeding, and convenience during therapy.^[18]^ However, more studies are warranted to prove rivaroxaban efficiency and safety in specific thrombosis-related conditions such heart failure or LVNC. Moreover, the ideal dose of rivaroxaban also warrants further consideration. Normally, the dose of rivaroxaban suggested for treating thrombosis, not prophylaxis, is >15 or 20 mg daily. In the present patient, only 10 mg of rivaroxaban was necessary for efficient thrombosis treatment, which suggests such a low dose might be enough for LVNC or early LVNC, in which the cardiac function as well as the blood flow within left ventricle are relatively preserved. Though our case is just one example, such an experience may inspire or imply a potential approach to treat thrombus in LVNC, a rare heart disease, before large clinical trials can be used to guide the therapy.

## 4 Conclusion

LVNC is a rare heart disease associated with obvious thromboembolic risk. In our case report, we have shown the success of low dose rivaroxaban in the anticoagulation therapy in LVNC patients. However, it is clear that the antithrombotic strategy for this disease requires more attention.

## Supplementary Material

Supplemental Digital Content

## Supplementary Material

Supplemental Digital Content
